# Identification of a Membrane Binding Peptide in the Envelope Protein of MHV Coronavirus

**DOI:** 10.3390/v12091054

**Published:** 2020-09-22

**Authors:** Entedar A. J. Alsaadi, Benjamin W. Neuman, Ian M. Jones

**Affiliations:** 1 School of Biological Sciences, University of Reading, Reading RG6 6AJ, UK; entedarali@ymail.com; 2 Department of Microbiology, College of Medicine, Thi-Qar University, Thi-Qar, Iraq; 3 Biology Department, Texas A&M University, Texarkana, TX 75503, USA; bneuman@tamut.edu

**Keywords:** coronavirus, envelope, E protein, peptide, membrane, GUV, expression

## Abstract

Coronaviruses (CoVs) are enveloped, positive sense, single strand RNA viruses that cause respiratory, intestinal and neurological diseases in mammals and birds. Following replication, CoVs assemble on intracellular membranes including the endoplasmic reticulum Golgi intermediate compartment (ERGIC) where the envelope protein (E) functions in virus assembly and release. In consequence, E potentially contains membrane-modifying peptides. To search for such peptides, the E coding sequence of Mouse Hepatitis Virus (MHV) was inspected for its amino acid conservation, proximity to the membrane and/or predicted amphipathic helices. Peptides identified in silico were synthesized and tested for membrane-modifying activity in the presence of giant unilamellar vesicles (GUVs) consisting of 1,2-dipalmitoyl-sn-glycero-3-phosphocholine (DPPC), sphingomyelin and cholesterol. To confirm the presence of membrane binding peptides identified in the context of a full-length E protein, the wild type and a number of mutants in the putative membrane binding peptide were expressed in Lenti-X-293T mammalian and insect cells, and the distribution of E antigen within the expressing cell was assessed. Our data identify a role for the post-transmembrane region of MHV E in membrane binding.

## 1. Introduction

The coronavirus envelope protein (E) is a small hydrophobic protein ranging from 74–109 amino acids [1,2]. It has an N-terminal domain, a long alpha helical transmembrane domain and a C-terminal hydrophilic domain, and is found incorporated into the virus particles of all coronavirus groups at low levels [3,4,5,6,7]. Two membrane topologies have been suggested for the E protein: hairpin or transmembrane [5,8]. E also interacts with the M protein, and mutants of M that are unable to bud from cells can be complemented by forms of E [9,10]. The membrane curving properties of E are such that the coexpression of M and E is adequate for the efficient formation of virus-like particles (VLP) [11,12], and these can also incorporate the S protein if it is coexpressed [13]. For many coronaviruses, including MHV, E protein also functions as an ion channel, a viroporin [14,15], affecting the trafficking of virions in the secretory pathways and membrane permeability, both of which are essential for virus growth [3,16,17]. While E function is critical for virus assembly, its viroporin activity in mobilizing calcium ions and its interactions with host cell tight junction proteins have also been implicated in the pathologies of some coronavirus infections [16,18]. An additional role for E in viral pathogenesis is an anti-apoptotic effect on host cells during virus replication [5]. The relative significance of the E encoded ion channel activity to virus morphogenesis, as opposed to its structural role in binding M, is uncertain. However, its role as a virulence factor has been demonstrated by many studies on SARS-CoV E protein which have shown altered pathogenesis in a mouse model, either by an effect of virus production and by effects on expressing cells [7,19,20]. Stimulated by the pandemic of SARS-CoV-2 [21], a recent protein interaction map of virus infected HEK cells identified host proteins of the vesicular sorting pathway and bromodomain proteins as interacting with E, the latter of which may have therapeutic potential [22]. Notwithstanding its multifunctional nature, the interaction of E with membranes is central to its biological role, and peptides with direct membrane binding properties are present within the coding region. The mapping of such peptides may help to interpret E mechanisms of action and offer the potential for inhibitor development. Here, we test E-derived peptides for membrane binding activity in vitro and confirm those identified as positive in the context of the full length protein expressed in two different cell types.

## 2. Materials and Methods

All chemical reagents were purchased from ThermoFisher Scientific unless otherwise stated, and were used following the vendor’s recommendations. Lipids were obtained from Avanti polar lipids. 

### 2.1. Peptide Synthesis

Peptides used for the in vitro analysis (Table 1) were derived from the E protein of MHV coronavirus (accession number AAU06359), synthesized and supplied as a lyophilized product with a stated mean purity of ≥70% by Cambridge Research Biochemicals, UK. Peptide stock solutions were prepared in a buffer consisting of 0.1mM sucrose, 0.1 mM glucose, 10 mM DTT and 0.5% (*v/v*) DMSO. A positive control peptide, the M2-influenza peptide, a well characterized amphipathic helix sufficient for budding into GUVs and the formation of large luminal vesicles (LUVs) [23], was used to validate the GUV assay. Negative controls were provided by buffer only in order to match test samples in all but the test peptide.

### 2.2. Giant Unilamellar Vesicles (GUVs)

GUVs were generated by electroformation using a Vesicle Prep Pro (Nanion Technologies GmbH, Munich, Germany) with a mix of 5 mM 1,2-dipalmitoyl-*sn*-glycero-3-phosphocholine (DPPC), 4 mM egg sphingomyelin and 0.5 mol% cholesterol, which were first dissolved in chloroform. To visualize the GUVs, 0.5 mol% of naphtha [2–alpha] pyrene (Tokyo Chemical Industry UK Ltd, Oxford, UK) was added. Lipids were mixed in an amber vial to a final total lipid concentration of 9 mM before preparation by electroformation. Briefly, 20µl of lipid stock was spread on the conductive side of an indium tin oxide slide (ITO-slide), air dried and put into a vacuum desiccator for 1 h to remove all solvent. A 28-mm O-ring was coated on one side with silicon grease and placed around the dried lipid film, which was then hydrated with 750 µL of 0.1 mM sucrose solution in deionized water. A second ITO slide was placed on top of the O-ring with the conductive sides facing, and a voltage of 3 V peak to peak at a frequency of 5 Hz was applied to the ITO slides over a period of 2 h at 50 °C [24]. GUVs were imaged using an EVOS-FL digital fluorescence microscope (EVOS, USA).

### 2.3. GUV Deformation Assay

Following electroformation, the chamber was disassembled and the sucrose solution removed to leave the GUVs attached to one ITO slide. Diluted peptide was added immediately at the desired concentration and the field was imaged 0, 1, 2 and 5 min thereafter. ImageJ was used to measure the shape and the relative size of the GUVs. Experiments were performed in triplicate and the average and standard deviation were calculated. Buffer only and M2-Influenza peptide controls were treated in the same way. GUV-peptide incubations were at room temperature. 

### 2.4. Statistical Analysis

Statistical significance was calculated using SPSS version 22, using Linear Mixed Model (LMM) (p•0.05). Results were expressed as mean ± SEM. Figures were generated using GraphPad Prism 7.0b software.

### 2.5. Cell Culture

Lenti-X 293T cells were cultured and maintained in Dulbecco’s modified Eagle medium (DMEM) (Sigma Aldrich) supplemented with 10% fetal bovine serum (FBS) (GE Healthcare) and 0.2% penicillin-streptomycin solution (Gibco/Invitrogen, Paisley, UK) at 37 °C with 5% CO_2_. *Spodoptera frugiperda* (Sf9) cells (Invitrogen) were maintained in EX-CELL 420 medium (Sigma, Gillingham, UK) supplemented with 2% fetal bovine serum and 1% penicillin/streptomycin solution at 27 °C with shaking.

### 2.6. Constructs and Mutagenesis

The coding sequence of MHV E (GenBank accession: AY700211.1) was amplified by PCR from cDNA kindly supplied by Dr. Volker Thiel using the primers HSV-E-FW (5’GCGCCATGGCACAGCCAGAACTCGCCCCGGAAGACCCCGAGGATTTTAATTTATTCCTTACAGACACAGTATGGTATGTGGGG-3’) and HSV-E-RV (5’-GCGCTCGAGGATATCATCCACCTCTAATAGGGG-3’) and the amplicon cloned into pTriEx 1.1 (Merck Millipore, Darmstadt, Germany) between the *Nco*I and *Xho*I restriction sites. To provide markers for the detection of expression by Western blot, the WT E coding region was cloned in frame with an upstream HSV and downstream His tag, both provided by the vector. Following initial expression experiments, detection of the HSV tag was found to be poor. As a result, mutant E sequences were synthesized de novo (Integrated DNA Technologies, Dresden, Germany) and cloned similarly, except they were in frame only with the downstream His tag. All constructs were confirmed by DNA sequencing prior to use. 

### 2.7. Immunofluorescence

One day before transfection, 1.25 × 10^5^ Lenti-X 293T cells (Takara, Saint-Germain-en-Laye, France) were seeded into 12 well plates containing a glass coverslip in each well and incubated for 24h at 37 °C/5% CO_2_. The following day, the cells were transfected using Lipofectamine 3000 transfection reagent (Invitrogen) with plasmids encoding wildtype or mutant MHV-E and incubated for 24h at 37 °C/5% CO_2_. A control vector pTriEx1.1-GFP expressing Green Fluorescent Protein (GFP) gene was used to visualize the efficiency of transfection, typically 20–30%. Twenty four hours after transfection, the media was removed and the cells were washed twice with cold PBS for 5 min, then incubated in fix and permeabilization buffers for 1 h at room temperature (eBioscience, San Diego, USA). Fixed and permeabilized cells were incubated with Anti-His–Alexa Fluor 488 conjugate Ab (4E3D10HH2/E3, ThermoFisher Scientific) for 1 h at room temperature diluted 1:100 in 1× permeabilization buffer, washed twice with TBS for 15 min each at room temperature in the dark, and then counterstained with DAPI. The fixed cells were mounted with a drop of Slowfade™ Gold reagent before being imaged using an EVOS-FL digital fluorescence microscope. Typical images were captured at 20× magnification and further manipulated, if required, using ImageJ software [25].

### 2.8. Baculovirus Expression

The flashBAC™ GOLD (FBG) baculovirus expression system (Oxford Expression Technologies, Oxford, UK) was used to produce recombinant baculoviruses. Small-scale protein expression was performed by infection in a 6-well plate seeded with 1 × 10^6^ *Sf*9 cells per well using 200 μL of a high titre stock of the recombinant baculovirus, typically passage 3, and incubated for 3 days at 27 °C. After incubation, cells were harvested and used for western blot with an anti-His antibody.

### 2.9. SDS-PAGE

Proteins were separated by SDS-PAGE using 4–12% precast Tris-Glycine SDS polyacrylamide gels (Invitrogen) for 30 min at 170V. After electrophoresis, gels were either stained by Coomassie blue R250 or transferred to PVDF membranes for Western blot analysis.

### 2.10. Western Blot

Following transfer to PVDF membranes (Whatman, Chalfont St. Giles, UK) using a semidry western blotting apparatus, membranes were incubated in blocking buffer (5% skimmed milk powder in TBST (0.2% Tween-20, 1× TBS)) for 1 h. Membranes were incubated with the primary antibody at 1:10,000 in 1× TBST buffer for 1 h, followed by three washes for 5 min each in TBST buffer, and subsequently, with a secondary horseradish-peroxidase (HRP) antibody conjugate (Dako, Glostrup, Denmark) diluted 1:10,000 in 1× TBST for 1 h followed by three washes for 5 min each with TBST buffer. The membrane was finally washed with TBS and the bound HRP activity was detected using chemiluminescence imagery on a Syngene G: BOX. 

### 2.11. Membrane Re-Probing

To allow reprobing of a PVDF membrane with a different antibody, the membrane was stripped using a stripping buffer (100 mM β-mercaptoethanol, 2% SDS, 62.5 mM Tris-Hcl pH 6.7) for 30 min at 50 °C. The membrane was then washed three times with TBST buffer, 10 min each, and then reblocked and probed as before. 

### 2.12. Differential Membrane Fractionation

A total of 2.5 × 10^6^ Sf9 cells were seeded in T25 flasks as monolayers, infected with recombinant baculoviruses at an MOI = 3 and incubated for 72 h at 27 °C. The infected cells were harvested by loosening the monolayer into the media and collected by centrifugation at 3000× *g* at 4 °C for 20 min. Cell pellets were resuspended in 500 µl of cold PBS and lysed by sonication for 10 m with on/off pulses at 20-s intervals (Sonics, Vibracell^TM^). The cell lysates were briefly clarified (1500× *g* for 5 min) and then centrifuged at low speed, i.e., 10,000× *g*, at 4 °C for 30 min to collect large membrane debris. The pelleted material was kept as a low-speed pellet (LSP). The collected supernatants were then centrifuged at high speed, i.e., 100,000× *g*, for 90 min at 4 °C in a Beckman TL-100 ultracentrifuge and the pellets were retained as high-speed pellets (HSP). Low- and high-speed pellets were tested for the presence of MHV E by western blot using an anti-His antibody, as before. 

## 3. Results

### 3.1. Bioinformatic Analysis of CoV-E Proteins

**Figure 1:**
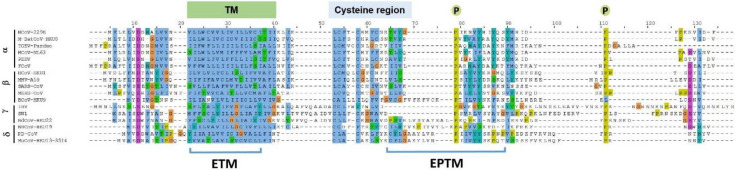
Multiple sequence alignment of coronavirus E protein. Alignment was done using Jalview 2.9.0b2 and the position of key features, TM, EPTM, Cys region and conserved prolines indicated. Four genera of coronavirus are represented as follows: α-CoV is represented by HCOV-229E, Human coronavirus 229E (NP_073554.1); M-BatCoV-HKU8, Miniopterus bat coronavirus Hong Kong University 8 (YP_001718614.1); TGEV-Purdue, transmissible gastroenteritis virus- Purdue (ABG89336.1); HCoV-NL63, Human coronavirus NL63 (YP_003769.1); PEDV, Porcine epidemic diarrhea virus (NP_598312.1); FCoV, Feline coronavirus (YP_004070197.1); β-CoV include HCoV-HKU1, Human coronavirus Hong Kong University 1(YP_173240.1); MHV-A59, Murine hepatitis virus-A59 (NP_068673.1); SARS-CoV, Severe acute respiratory syndrome coronavirus (NP_828854.1); MERS-CoV, Middle East respiratory syndrome coronavirus (YP_009047209.1); BCoV-HKU9, Bat coronavirus Hong Kong University 9 (YP_001039973.1); γ-CoV include IBV, Infectious bronchitis virus (ADP06512.1); SW1, sperm Whale coronavirus 1(YP_001876438.1); BdCoV-HKU22, Bottlenose dolphin coronavirus Hong Kong University 22 (AHB63482.1); δ-CoV consists of NHCoV-HKU19, Night-heron-coronavirus- Hong Kong University 19 (AFD29227.1); PD-CoV, Porcine coronavirus Hong Kong University 15 (YP_005352832.1); MCoV-HKU13, Munia coronavirus Hong Kong University 13-3514(YP_002308507.1). TM: transmembrane domain; highly conserved cysteine residues indicated; conserved proline indicated by stars. Blue represents hydrophobic amino acids (A, I, L, M, F, W, V); Red represents positive charge amino acids (K, R); Magenta represents negative charge amino acids (E, D); Green represents polar amino acids (N, Q, S, T); Pink represents cysteines (C); Orange represents glycines (G); Yellow represents prolines (P); Cyan represents aromatic amino acids (H, Y); White represents any unconserved/gap. The SARS-CoV-2 E protein is 95% identical to SARS E, so was not included as a distinct entry.

Several viral proteins encode amphipathic helices that modulate membrane curvature, e.g., the M2 protein of the influenza virus and the nonstructural protein 4B of the Hepatitis C virus [23,26,27]. The program Amphipaseek [28] was used to assess local amphipathy and putative membrane-binding regions for a phylogenetically diverse set of E proteins representing 17 CoVs, and the output was visualized using Jalview V 2.9.0b2 [29]. CoV E proteins have related topologies but otherwise limited direct homology outside of the clearly predicted TM domain. However, amphipathicity occurred in a region immediately downstream of the TM domain, a region which contains the previously mapped cysteine region [30] at the end of the predicted transmembrane domain (Figure 1) and which is also palmitoylated intracellularly [8,31] to facilitate contact with the lipid bilayer [32]. In addition, two conserved proline residues occur in this region, plausibly providing conformational flexibility that may be important for E-E interactions or between E and other proteins [8,33]. Spanning the first conserved proline, a 15 residue sequence, i.e., 50–64 in MHV A59 E, including a number of hydrophobic residues, was evidently relatively well conserved among most α and β CoVs, and was also shared with the more distantly related γ and δ CoVs, with the exception that insertions containing further multiple hydrophobic residues were also present (Figure 1). Based on these criteria this peptide, the E post TM peptide (EPTM), was considered to have potential for direct membrane interaction.

### 3.2. Effect of MHV-EPTM Peptide on Shape and Size of GUVs

**Figure 2:**
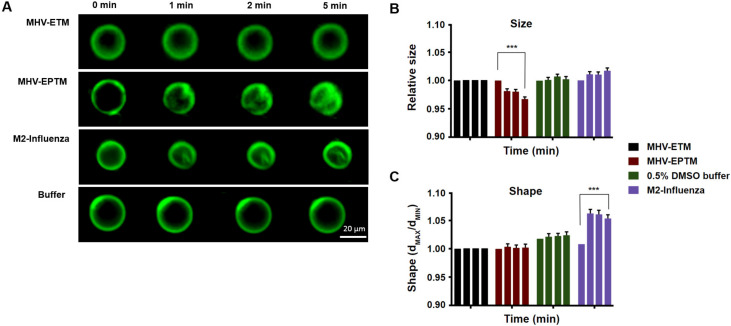
Effect of MHV E protein-derived peptides on size and shape of GUVs. (**A**) Fluorescent images of electroformed GUVs treated with peptides MHV-EPTM, MHV-ETM and M2-Infleunza and imaged at 0, 1, 2 and 5 min postaddition. (**B**) GUV relative size estimated by Ramanujan’s first approximation. Then standard deviation for both long and short measurements for a vesicle was also measured and averaged for each GUV for three separate experiments of 40 GUVs each. (**C**) GUV shape, measured as the ratio between the longest and shortest radii and averaged for each GUV for three separate experiments of 40 GUVs each. The scale bar indicates 20 µm. Error bars shown are mean ± SEM. The stars *** indicate significance (*p* ≤ 0.001; with respect to the corresponding buffer; Linear Mixed). Each coloured group of four columns represents the data for a single peptide test at each time point. Left to right, 0, 1, 2 and 5 min. Some error bars are too small to be observed.

To address its potential as a membrane active peptide, the 15 residue wild type peptide was synthesised and reconstituted with giant unilamellar membranes (GUVs), and the effect on size and morphology were measured. In addition, peptides ETM, representing the E protein TM domain itself, and the M2 influenza peptide were tested (Table 1). GUVs, composed of 5 mM DPPC, 4 mM egg SM and 0.5 mol% cholesterol were reconstituted with the peptides at a concentration of 10 µM and the GUVs were measured at 1, 2 and 5 min thereafter by fluorescence microscopy, as described elsewhere [34]. Shape was measured as the ratio between the longest and shortest radii, while relative GUV size was estimated by Ramanujan’s first approximation, taking an average of 40 GUVs per experiment for three separate experiments. Peptide EPTM changed the size but not the shape of the GUVs membrane, leading to membrane deformation that increased with time, while GUVs treated with buffer only showed no deformation (Figure 2). Interestingly, peptide ETM showed no effect on either the shape or size of the GUVs. Interaction of MHV-EPTM peptide with the GUV membranes made the GUVs smaller in size and more rugged in appearance, which was possibly indicative of fragmentation of the lipid bilayers after peptide insertion. The lack of effect by the ETM peptide suggested that this peptide alone is not membrane active and may need to be presented in the context of the complete E protein for biological activity. The assay was validated by use of the M2-influenza peptide which showed a statistically significant change in GUV shape with the formation of intraluminal-vesicles (ILVs) as described [23], although no significant change in size was apparent (Figure 2).

### 3.3. Expression and Distribution of MHV E and EPTM Mutants in Mammalian Cells

**Figure 3:**
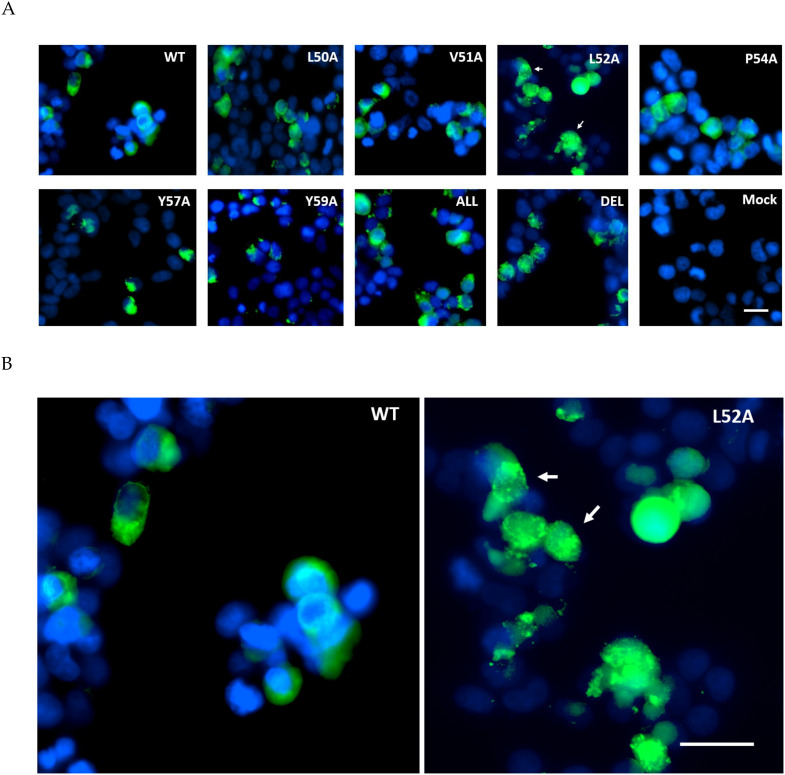
Immunofluorescent staining of MHV E protein expression in Lenti-X 293T cells. (**A**) Cells were transfected with pTriEx1.1 vectors encoding WT MHV-CoV E or various alanine mutants, fixed and permeabilized and detected with anti-His Ab conjugated to Alexa Flour 488 (green). Nuclei were counterstained with DAPI (blue). Punctate staining is indicated. (**B**) Enlarged images of the WT and L52A panels with punctate staining indicated. In both panels the scale bar is 20 M.

To validate the EPTM peptide sequence as membrane active in the context of the full length E protein, E was expressed in both mammalian and insect cells through the use of a dual promoter vector, pTriEx1.1, which permits expression from the same vector in both systems. MHV E protein was expressed as the wild type protein and also as a series of alanine scanning mutants in which conserved and hydrophobic residues were exchanged for alanine (Table 2). In addition, the entire region was deleted within the E coding sequence. Mammalian expression was done by transfection of Lenti-X 293T cells where expression is driven by the vector resident CAG promoter, and the expressed E protein was detected by immunofluorescence imaging with an anti His tag antibody 24 h post-transfection. The WT MHV E protein was found to be distributed evenly throughout the cytoplasm with a slight concentration near the nucleus, plausibly the Golgi body. In all the alanine scanning mutants of E, expression levels were similar with little diminution of the overall signal, but in most cases, the pattern of staining was altered to a more granular punctate appearance (Figure 3). This was particularly notable for mutation L52A, where almost all of the fluorescence signal was associated with a punctate distribution. This data suggests that the EPTM sequence, identified as causing membrane association in an isolated peptide, also influences the behaviour of the complete protein in a physiologically relevant environment.

**Figure 4:**
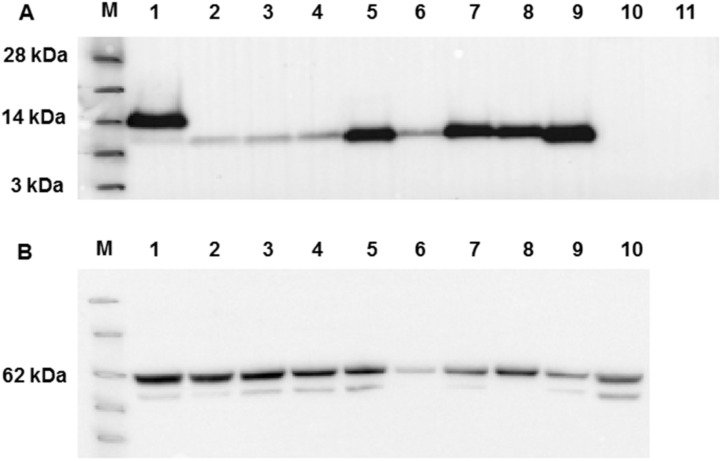
Western blot analysis of WT MHV-E protein expression and mutants following expression in insect cells. (**A**) Western blot using anti-His antibody. Lane M - See Blue™ Plus2 Pre-Stained Protein Standard (Invitrogen). Lanes 1-11 are samples as follows: 1: WT E, 2: L50A, 3: V51A, 4: L52A, 5: P54A, 6: Y57A, 7: Y59A, 8: all mutants, 9: deleted EPTM, 10: GFP baculovirus (positive control), 11: mock. (**B**) Western blot by anti baculovirus surface glycoprotein gp64 following stripping of the membrane used for panel A Key molecular mass markers are indicated to the left of each panel.

To provide a more quantifiable measure of membrane association, a more productive protein expression system, i.e., expression in insect cells via the construction of recombinant baculoviruses, was investigated with the same panel of E variants. Following the generation of recombinant viruses, insect cells were infected at high MOI and their ability to express E protein assessed by Western blot at 3 days postinfection when synthesis from the vector encoded P10 promoter was maximal. To facilitate detection, the WT E protein was expressed with both HSV and His tags, at the N- and C-termini respectively, a predicted molecular mass of ~13 kDa. Of these two tags, however, only probing with the His antibody detected the expressed product consistently. As a result, all mutant E proteins were expressed with only the C-terminal His tag, a predicted molecular mass of ~11 kDa. All E proteins were expressed at the molecular weight expected, but the expression level varied with mutation (Figure 4A). Half of the mutants expressed at levels similar to the wild type, but mutations L50A, V51A, L52A and Y57A were present at reduced levels, consistent with a potential role of these residues in protein folding and stability. To ensure the levels of infection were equivalent, the blot was stripped and reprobed with a monoclonal antibody to the major baculovirus glycoprotein, gp64, a marker for infection level, which showed near equivalent infection in all cases (Figure 4B). 

### 3.4. Differential Membrane Fractionation

**Figure 5:**
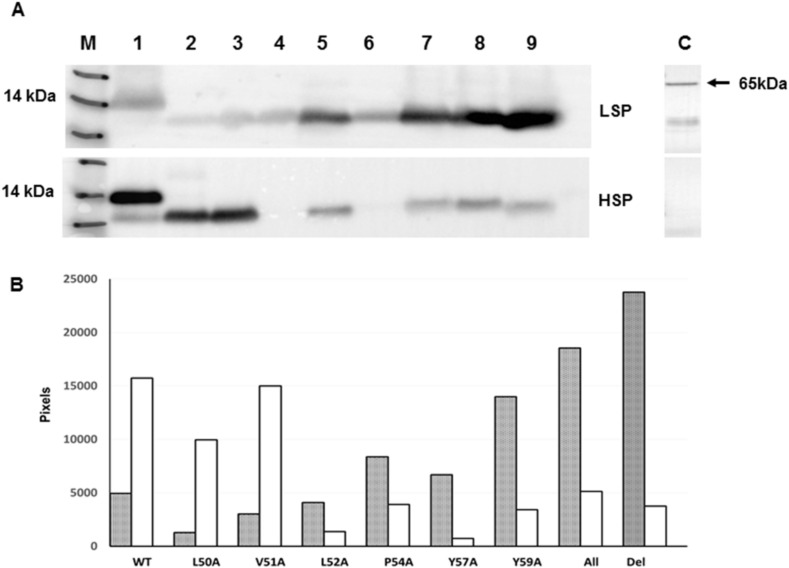
Western blot Analysis of WT MHV-E and mutants following membrane partition by differential centrifugation. (**A**) Detection of E protein by anti-His antibody in the low- and high-speed membrane fractions (marked). Lane M - See Blue™ Plus2 Pre-Stained Protein Standard (Invitrogen) with 14KDa marker identified. Lanes 1-9 are samples as follows: 1: WT E, 2: L50A, 3: V51A, 4: L52A, 5: P54A, 6: Y57A, 7: Y59A, 8: all mutants, 9: deleted EPTM. The single panel to the right of panel **A,** marked **C,** is the LSP and HSP fractions blotted with an antibody to calnexin. (**B**) The altered distribution of E, dependent on its sequence, is evident by the relative band intensity and was measured by ImageJ densitometry. The filled bars are the LSP samples, the open bars the HSP.

To investigate whether the mutations introduced into MHV-E resulted in an effect on cellular localization in more detail, plausibly by altered membrane association, infection of insect cells with each recombinant virus was repeated on a larger scale and the infected cells harvested at 2 days postinfection. The cells were lysed by sonication in the absence of detergent, clarified and subjected to differential membrane fractionation to produce low-speed (LSP) and high-speed membrane pellet (HSP) fractions. To confirm the fractionation, LSP and HSP samples were probed with an antibody to calnexin, an ER marker, which was largely found in the LSP fraction (Figure 5). The expression of the WT MHV E was found in both fractions, but was more strongly associated with the HSP fraction (Figure 5A), consistent with its known primary localization in MHV-CoV infected cells [35]. Similarly, mutations L50A and V51A partitioned mainly into the HSP fraction, although expression levels overall were reduced. Strikingly, the “all” mutant, in which all targeted residues were mutated to alanine, and the “del” mutant, in which the target peptide was deleted from MHV E, were found almost exclusively in the LSP fraction, despite high levels of expression. The remaining mutants carrying mutations L52A, P54A, Y57A and Y59A also associated preferentially with the LSP, although, as before, expression levels were lower in some cases, notably L52A and Y57A. Relative densitometry of the HSP and LSP bands revealed significant differences among the mutants with regard to their localisation to the different membrane fractions of E expressing insect cells (Figure 5B) and confirmed a role for the amphipathic MHV CoV E 50-64 peptide in membrane interaction.

## 4. Discussion

**Figure 6:**
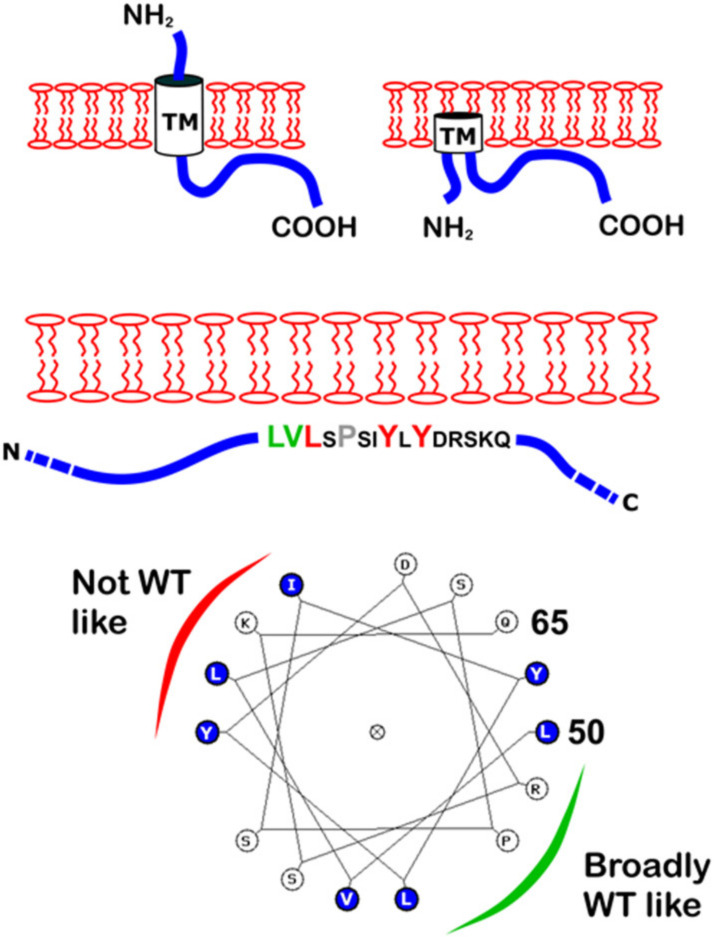
Predicted topological models of E and the EPTM peptide. **Above**: Two of the suggested topologies of E are shown with TM, N- and C-termini indicated. **Middle**: The EPTM peptide is shown within the C-terminal region. Mutated residues are enlarged and coloured according to the level of membrane repartitioning observed. **Below**: A helical wheel depiction of the EPTM peptide with a suggested interpretation of sidedness with respect to membrane attachment.

The CoV E proteins consist from a short hydrophilic amino terminal region, a hydrophobic transmembrane region and a carboxy-terminal region that encompasses the majority of the protein [30]. The E proteins are multifunctional proteins involved in virus assembly and release, and have been located in the ERGIC and Golgi of infected cells but do not traffic to the infected cell surface [35]. As studies of E have often involved different coronaviruses and different experimental systems, the unambiguous basis of this localisation has not been described. An amphipathic helix, EPTM, detected in the post-TM region of E, was suggested by bioinformatics analysis and assessed for direct membrane interaction in vitro by binding to GUVs. For comparison, the predicted E protein TM domain, ETM, and an established membrane active peptide from the influenza M2 protein were also included. EPTM, but not ETM, caused a change in GUV size and appearance, consistent with direct membrane binding. Following expression of the complete E protein with mutations in the same identified peptide, altered membrane binding in two distinct cell types, mammalian and insect, was apparent. These data support the hypothesis that the membrane binding observed for the EPTM peptide in vitro occurs also in the context of the complete E protein expressed in a physiologically relevant environment. CoV E protein is a known integral membrane protein, and it has been proposed that the basis of membrane interaction is by one of a number of possible topologies, e.g., as a type III membrane protein, as a membrane hairpin or as a glycosylated type II membrane protein [5]. The expression of E in insect cells resulted in a single band detected by western blot that did not have the appearance typical of a glycosylated protein. Moreover, in the latter topology, the EPTM region is predicted to be free on the luminal side of the membrane and not to interact with it. The data here are inconsistent with this prediction and are more explicable by assuming that either the type III membrane protein or the membrane hairpin topology is more likely. In both cases, it is postulated that the post-TM region folds back against the membrane, where it may be additionally anchored by cysteine palmitoylation, although the precise mechanism of interaction remains unknown. Assuming that the predominant punctate staining in mammalian cells represents misfolded E protein that induced stress granules, then mutants L52A, Y57A and Y59A formed a group with a shared phenotype. The “all” mutant, which necessarily included these mutations, was also punctate in appearance. By contrast, L50A and V51A were more similar to the WT straining pattern. Biochemical fractionation of membrane from insect cells following the construction and validation of recombinant baculoviruses broadly recapitulated this division. The relative level of E protein following differential centrifugation of the cell membranes showed that WT, L50A and V51A mutants were predominantly associated with the HSP fraction with a minority in the LSP fraction (31.4%, 12.7 % and 20.2% respectively), while the remaining mutants were predominantly associated with the LSP fraction. Some mutants, notably L52A and Y57A, were expressed at lower levels but were hardly present in the HSP faction. As the WT exhibited an HSP pattern, we interpret this to be authentic partitioning into membranes, whilst the LSP pattern is likely to be aggregated protein trapped in the ER by virtue of its inability to associate with membranes correctly. Despite its conservation, the role of the P54A mutant was equivocal, i.e., some way between the two extremes but tending towards non-WT-like mutants. These data suggest a possible model for EPTM interaction with the membrane in which the C-terminal hydrophobic residues within the 15 residue region are the most relevant. Interestingly, these fall broadly on one side of the helix prediction for EPTM, while those broadly tolerated fall on the other (Figure 6). Plausibly, this distinction relates to membrane binding verses the requirement for E to also bind other ligands. Further work will be required to address the level of membrane curvature afforded by this interaction and the extent to which it relates to the various functions reported for CoV E, such as reorientation of the plasma membrane within the Golgi [33], interaction with the M protein [3,36,37] and viral particle scission [38,39]. In SARS CoV, E residues which reportedly bind to the membrane include Valine-52, Tyrosine-59 and Lys-63 [40]. These residues are fully conserved in SARS-CoV-2 within an overall E similarity of 98%, and are also equivalent in the MHV E peptide identified here. Membrane binding by these residues in SARS E is thus supported by the observations made here for V52A and Y59A, although Lys 62 was not examined. E has been considered a therapeutic target for SARS in relation to its viroporin function which is linked to inflammation [41], and more recently, for SARS-CoV-2 as a result of its mapped interactions with bromodomain proteins, for which drugs already exist [21]. A more detailed mapping of membrane binding by E and how it functions once bound may facilitate future drug discovery programs targeting this small, variable but multifunctional protein.

## Figures and Tables

**Table 1:** Peptides used for in vitro GUV deformation analysis.

Peptide No.	Name	Residues	Sequence
1	MHV ETM	16–30	IIFIFAVCLMVTIIV
2	MHV EPTM	50–64	LVLSPSIYLYDRSKQ
3	M2-Influenza	44–62	RLFFKCIYRFFEHGLKRG

**Table 2:** Alanine scanning mutations.

Name	Sequence
WT	LVLSPSIYLYDRSKQ
L50A	**A** VLSPSIYLYDRSKQ
V51A	L **A** LSPSIYLYDRSKQ
L52A	LV **A** SPSIYLYDRSKQ
P54A	LVLS **A** SIYLYDRSKQ
Y57A	LVLSPSI **A** LYDRSKQ
Y59A	LVLSPSIYL **A** DRSKQ
All	**AAA** S **A** SI **A** L **A** DRSKQ
Del	----------------------------

## Abbreviations

MOI	multiplicity of infection
HSV	Herpes Simplex Virus
His	polyhistidine
MHV	Mouse Hepatitis Virus
GUV	Giant Unilamellar Vesicles
